# Influence of pre-exposure time on the toxicities of different temperature effect insecticides to *Apolygus lucorum* (Hemiptera: Miridae)

**DOI:** 10.1371/journal.pone.0272429

**Published:** 2022-08-15

**Authors:** Ya’nan Dou, Jingjie An, Xiu Yan, Zhihong Dang, Jianglong Guo, Zhanlin Gao, Yaofa Li

**Affiliations:** Plant Protection Institute, Hebei Academy of Agricultural and Forestry Sciences/IPM Center of Hebei Province/Key Laboratory of Integrated Pest Management on Crops in Northern Region of North China, Ministry of Agriculture and Rural Affairs, P. R. China, Baoding, China; Institut Sophia Agrobiotech, FRANCE

## Abstract

Temperature can have influences on the toxicities and efficacies of insecticides. Therefore, it is important to accurately evaluate the temperature effect (TE) on the toxicities of insecticides to insects. Previous studies have shown that the pre-exposure of insects to temperatures before their contact with insecticides, caused variations in their toxicities. However, most of these studies focused on the TE of the insecticides post-treatment. In this study we hypothesized that pre-exposure time of insect at different temperature can influence the toxicities of insecticides. We then evaluated the influence of different pre-exposure time (0, 2, 4, 8, 12 and 24 h) on toxicities of three different temperature effect insecticides (TEIs) to *Apolygus lucorum* at 15, 25 and 35°C respectively. We found that all toxicities of three TEIs to *A*. *lucorum* did not vary with pre-exposure time at 25°C. The LC_50_ of hexaflumuron (positive TEI) only decreased (from 1800.06 to 237.40 mg/L) at 15°C, with an increase in the pre-exposure time. Whereas the LC_50_ of *β*-cypermethrin (negative TEI) decreased from 225.43 to 60.79 mg/L at 35°C. These results also showed that the temperature coefficients (TCs) of the toxicities were influenced by pre-exposure time at different temperatures. For hexaflumuron, all the TCs at 25°C and 35°C decreased, as the pre-exposure time increased. For *β*-cypermethrin, the TCs decreased significantly only at 35°C. The toxicity and TCs of phoxim (non-effect TEI) showed no obvious fluctuation at the tested temperatures. These results showed that when the pre-exposure times were extended, the toxicities of the positive / negative TEI showed an increase at the temperature where the pest was less sensitive to the insecticides. These results can be applied to determine the toxicities / bioactivities of different insecticides accurately at different temperatures.

## Introduction

The toxicities (or bioactivities) of insecticides to insects, depend on the physical and chemical properties of the insecticides and the physiological metabolic activities of insects [1]. Ambient temperature, humidity, light, and pH are the main physical factors that modify these interactions [2, 3]. The effects of temperature on the toxicities of insecticides are quite complex and varied, so the study of the correlation between these, is important for the scientific and rational development and use of insecticides [4]. The increase or decrease in insecticide toxicity with temperature increases, is termed ‘positive’ or ‘negative’ temperature effect (TE) respectively, and ‘non-effect’ if the toxicity is unaffected by temperature increase. Temperature effect is widespread and has been reported in many related studies. Different insecticides show different degrees of TE in their toxicities to different insect species and even within the same species. For example, diamide insecticides showed varying degrees of positive TEs in their toxicities to lepidopteran insects at 15°C-35°C [5]. Also, the toxicities of cycloxaprid, nitenpyram, triflumezopyrim and chlorpyrifos against *Nilaparvata lugens* all showed obvious positive TE, while etofenprox showed a negative TE [6].

The relationship between temperature and insecticides has become an important research area in IPM [4]. When insecticides are applied, positive or negative temperature effect insecticides (TEIs) can achieve better control effect with less dosage at their respective sensitive temperature conditions. Additionally, it can also effectively reduce pesticide residues and the harm to beneficial arthropods. However, this requires an initial evaluation of the effect of temperature on the toxicity of an insecticide to an insect. For example, the rearing temperature of *Plutella xylostella* was reported to have positively correlated with the toxicity of deltamethrin and Bt Cry1Ac against the larvae [4]. Also, a short-term pre-exposure to a temperature of 45°C for at least 2 h significantly influenced the susceptibility of *Frankliniella occidentalis* adults to insecticides [7]. Besides, the level of methamidophos resistance in the progenies of resistant field population of *P*. *xylostella*, declined sharply when reared at high temperatures for one generation [8]. These studies all showed that pre-exposure of insects to different temperatures before contact with insecticides, caused variations in their toxicities. However, most of these studies focused on the TE variation on the toxicities of the insecticides after treatment [9, 10]. Also in some studies, insects were allowed to adapt to a given temperature for hours before exposure to chemicals, but the effect of the pre-exposure temperature on the toxicities of insecticides was rarely evaluated.

The mirid bug, *Apolygus lucorum*, acquired pest status gradually in northern China, and characterized by a wide temperature range (15°C–35°C) [11]. We have determined the toxicities of different insecticides to *A*. *lucorum* at different temperatures [12]. Because pre-exposure to a temperature can affect the toxicities of insecticides, we posed the hypothesis that the pre-exposure time of the tested insect at different temperature will also influence the toxicities of insecticides. We tested our hypothesis by determining the toxicities of three TEIs to *A*. *lucorum* with different pre-exposure time (0, 2, 4, 8, 12, 24 h) at 15, 25, 35°C respectively. The results lay a foundation for the design of efficient laboratory toxicity experiments and bioassays. They also provide a technical guidance for the field control of *A*. *lucorum* under different temperatures.

## Materials and methods

### Insects

The samples of *Apolygus lucorum* used in this experiment were obtained from a colony kept in the Plant Protection Institute, Hebei Academy of Agriculture and Forestry Sciences. The insects were not exposed to any insecticides during their continuous culturing in the laboratory over the years. They were reared on fresh asparagus bean pods (*Vigna unguiculata* L. Walp.) in a cylindrical plastic casing (12 cm in diameter by 10 cm in height). All larvae, adults, and eggs were held at 25±1°C, 60±5% relative humidity (RH), and under a 16:8 h light/dark cycle. As the physiological state of the third instar larvae is relative stable during the experiment period, the third-instar larvae were used in these experiments.

### Insecticides

Three TEIs of technical grade were used in this bioassay, and the TEs have been demonstrated in Liuʼs work [12]. Hexaflumuron, positive TEI, (97.0%) was provided by Hebei Yuze Chemical Technology Co., Ltd. *β*-cypermethrin, negative TEI, (96.3%) and phoxim, non-effect TEI, (92.5%) were all provided by Hebei Veyong Bio-Chemical Co., Ltd. The insecticides were dissolved in pure acetone, stored at 4°C and used within 2 days to prepare different dilution gradients (6–8 ratios). Test concentrations and controls for each insecticide were prepared in distilled water plus 0.1% Tween 80.

### Treatments

A series of experiments were carried out in controlled-temperature chambers. The temperatures in the chambers were set to 15, 25 and 35°C (±0.5°C). Insects were transferred from the rearing temperature of 25°C to 15°C and 35°C to acclimate for 0, 2, 4, 8, 12, 24 hours before being exposed to the insecticides. Fresh asparagus bean pods without any insecticides were twisted into circles with diameters of 5 cm, dipped into the appropriate insecticide solution for 30 s and then placed on absorbent paper to dry. Then asparagus bean pods were each placed in a plastic casing and 20 larvae were transferred onto each treated pod gently using a fine brush. Treatments with each insecticide ratio and controls were replicated three times (*n* = 60 larvae per concentration). Plastic casings with insects were maintained in temperature control chambers at 15, 25, or 35°C and 60±5% RH under a 16:8 h light/dark cycle. The mortality of *A*. *lucorum* was recorded for all treatments after 72 h. The insects were considered dead if they were completely unresponsive to slight touches with a brush.

### Data analysis

The mortality data was analyzed using the DPS v6.55 software to obtain the confidence limits and slopes of corresponding regression curve equation and the lethal concentration of 50% (LC_50_) under each treatment. Differences in LC_50_ values were considered significant if the 95% CLs did not overlap. Temperature coefficients (TCs) were calculated as the absolute value of the ratio of LC_50_ value at higher temperature to lower temperature. The TCs were marked with “+” in front, if the LC_50_ value decreased with an increase in temperature; otherwise, they were marked with “-” [13].

## Results

The pre-exposure time of *A*. *lucorum* to temperatures had varying effects on the toxicities of the different TEIs at 15°C and 35°C. For hexaflumuron (positive TEI), the toxicity increased sharply from 1800.06 mg/L (LC_50_ value, the same below) to 237.40 mg/L, with extension of the pre-exposure time from 0 h to 24 h at 15°C. But it increased inconspicuously at 35°C (Table 1). For *β*-cypermethrin (negative TEI), the reverse results were recorded. At 15°C, the toxicity of *β*-cypermethrin to *A*. *lucorum* had no obvious fluctuation with increase in the pre-exposure time from 0 h to 24 h, whereas it decreased significantly from 225.43 mg/L to 60.79 mg/L at 35°C (Table 2). For phoxim, (non-effect TEI), the toxicity fluctuated indistinctively with increase in pre-exposure time at both 15°C and 35°C (Table 3).

**Table 1:** Influence of pre-exposure time on the toxicity of positive temperature effect insecticide hexaflumuron to *A*. *lucorum* at 15, 25, 35°C.

Temperature (°C)	Pre-exposure time (h)	Probit analysis parameters					
		Intercept	Slope±SE	LC_50_	95% CL	*P*	TC
				(mg/L)			
15°C	0	2.34	0.82±0.22	1800.06	619.13–44664.32	0.36	-
	2	3.30	0.54±0.19	1459.98	428.22–91552.07	0.98	-
	4	2.97	0.66±0.21	1237.15	423.31–83635.79	0.98	-
	8	3.01	0.72±0.19	603.53	290.75–4373.98	0.45	-
	12	2.35	1.07±0.21	294.76	192.51–641.96	0.81	-
	24	2.66	0.98±0.18	237.40	160.65–458.90	0.41	-
25°C	0	3.51	1.17±0.20	18.69	9.04–28.61	0.23	+96.31*
	2	3.56	1.26±0.21	13.95	6.96–20.91	0.17	+104.66*
	4	4.00	0.95±0.20	11.18	3.63–19.23	0.15	+110.66*
	8	3.34	1.22±0.19	22.81	14.03–31.46	0.74	+26.46*
	12	3.73	0.99±0.18	19.26	9.53–28.88	0.91	+15.30*
	24	3.93	0.94±0.19	13.83	5.32–22.52	0.34	+17.17*
35°C	0	-0.31	3.83±0.51	24.39	20.23–32.46	0.29	+73.80*
	2	2.27	2.02±0.31	22.21	17.35–31.83	0.03	+65.74*
	4	0.61	3.31±0.43	21.16	17.74–27.21	0.05	+58.47*
	8	3.19	2.24±0.30	6.39	4.52–8.16	0.02	+94.45*
	12	1.31	3.60±0.50	10.63	8.82–12.49	0.38	+27.73*
	24	2.49	2.58±0.30	9.38	7.58–11.25	0.04	+25.31*

Note

* indicates that the toxicity at each temperature is significantly different from that at 15°C (*P*<0.05); TC indicates that temperature coefficient.

**Table 2:** Influence of pre-exposure time on the toxicity of negative temperature effect insecticide *β*-cypermethrin to *A*. *lucorum* at 15, 25, 35°C.

Temperature (°C)	Pre-exposure time (h)	Probit analysis parameters					
		Intercept	Slope±SE	LC_50_	95% CL	*P*	TC
				(mg/L)			
15°C	0	3.89	0.92±0.20	15.97	8.34–69.16	0.81	-
	2	4.04	1.02±0.19	8.85	5.59–20.94	0.80	-
	4	4.16	0.96±0.19	7.49	4.76–17.62	0.38	-
	8	3.82	0.95±0.20	17.34	8.94–76.54	0.89	-
	12	3.69	1.16±0.21	13.47	7.93–37.60	0.10	-
	24	3.95	1.08±0.19	9.40	5.96–21.81	0.06	-
25°C	0	2.70	1.54±0.25	30.97	1.33–58.66	0.43	-1.94
	2	3.28	1.17±0.28	29.61	17.79–89.22	0.69	-3.35
	4	3.66	0.97±0.23	24.42	14.42–72.17	0.78	-3.26
	8	3.46	1.03±0.27	31.59	17.88–126.48	0.91	-1.82
	12	2.55	1.61±0.30	33.34	22.09–72.03	0.70	-2.48
	24	4.14	0.68±0.22	18.63	9.76–64.77	0.24	-1.98
35°C	0	1.37	1.54±0.38	225.43	104.69–1826.06	0.68	-14.12*
	2	1.36	1.60±0.39	190.64	103.83–965.80	0.46	-21.54*
	4	2.29	1.34±0.22	107.06	69.29–230.51	0.30	-14.29*
	8	2.32	1.44±0.30	73.12	44.88–213.52	0.11	-4.22*
	12	-0.38	2.74±0.48	91.93	68.57–159.16	0.073	-6.82*
	24	3.47	0.86±0.26	60.79	33.78–399.10	0.38	-6.47*

Note

* indicates that the toxicity at each temperature is significantly different from that at 15°C (*P*<0.05); TC indicates that temperature coefficient.

**Table 3:** Influence of pre-exposure time on the toxicity of non-effect temperature effect insecticide phoxim to *A*. *lucorum* at 15, 25, 35°C.

Temperature (°C)	Pre-exposure time (h)	Probit analysis parameters					
		Intercept	Slope±SE	LC_50_	95% CL	*P*	TC
				(mg/L)			
15°C	0	0.38	1.71±0.42	504.83	254.34–3187.51	0.65	-
	2	2.02	1.05±0.24	702.41	287.42–6760.22	0.80	-
	4	1.95	1.04±0.25	856.07	320.45–12102.58	0.76	-
	8	1.64	1.31±0.25	375.77	204.08–1304.61	0.15	-
	12	1.52	1.27±0.31	538.42	234.97–4972.33	0.99	-
	24	0.99	1.71±0.26	219.79	147.03–436.53	0.06	-
25°C	0	2.12	1.09±0.23	445.28	256.05–1454.02	0.37	+1.13
	2	2.78	0.83±0.23	483.96	223.91–4462.14	0.47	+1.45
	4	3.19	0.66±0.21	545.84	241.23–10805.49	0.77	+1.57
	8	3.26	0.63±0.21	575.64	244.27–17566.57	0.99	-1.53
	12	1.69	1.18±0.25	636.61	335.44–2708.32	0.92	-1.18
	24	2.89	0.88±0.23	247.06	148.33–684.81	0.10	-1.12
35°C	0	0.37	1.70±0.32	521.35	342.79–1201.52	0.12	-1.03
	2	-0.92	2.49±0.37	234.63	188.72–320.84	0.14	+2.99
	4	-1.75	2.93±0.42	202.38	167.69–259.99	0.87	+4.23
	8	-1.02	2.54±0.38	231.43	188.84–311.33	0.07	+1.62
	12	1.36	1.42±0.28	366.02	254.99–725.81	0.44	+1.47
	24	1.05	1.81±0.22	150.24	121.27–195.88	0.21	+1.46

Note: * indicates that the toxicity at each temperature is significantly different from that at 15°C (*P*<0.05); TC indicates that temperature coefficient.

The pre-exposure time at different temperatures influenced TCs in the toxicities of different TEIs to *A*. *lucorum* accordingly. There was a significant increase in the toxicity of hexaflumuron as the temperatures increased (15°C, 25°C and 35°C). This showed that it was a strong positive TEI to *A*. *lucorum*. The TCs at 25°C and 35°C decreased as the pre-exposure time extended from 0 h to 24 h and was more significant at 25°C (Table 1). On the other hand, there was a significant decrease in the toxicity of *β*-cypermethrin as the temperatures increased, especially at 35°C. This showed that it was a strong negative TEI to *A*. *lucorum*. The TCs at 35°C decreased significantly as the pre-exposure time extended (Table 2), but showed no obvious fluctuation at 25°C. For phoxim, a weak positive TE (the highest TC value is +4.23) was only shown at 35°C under the 4 h pre-exposure treatment (Table 3).

## Discussion

Hexaflumuron has been shown to be a positive TEI to *A*. *lucorum* and *β*-cypermethrin to be a negative TEI, whereas the toxicity of phoxim was not affected by temperature significantly [12]. Results of the toxicities of three different TEIs to *A*. *lucorum* at 25°C showed that there was no significant variation in the pre-exposure time from 0 h to 24 h. This indicated that the physiological development within 24 hours did not change the susceptibility of *A*. *lucorum* to the three different TEIs insecticides at 25°C. Similarly, results from a previous study showed that a mild increase in duration, did not significantly change the life history traits of insects [14]. Nevertheless, they reported that the short mild episode in hot season or short hot episode in mild season observed in their study may have an impact on small insect populations [14]. We also made similar observations in this study. The susceptibility of *A*. *lucorum* to hexaflumuron and *β*-cypermethrin showed variations when the insects were pre-exposed at 15°C and 35°C. It was significantly higher at 15°C for hexaflumuron (positive TEI), but higher at 35°C to *β*-cypermethrin (negative TEI), when the pre-exposure time extended. These variations in susceptibilities produced different results to different TEIs, but commonly they all occurred at the temperature where the pest was less sensitive to the insecticides. Phoxim showed a weak positive temperature effect after 4 h pre-exposure treatment at 35°C, although it has been showed to have a non-effect temperature effect on *A*. *lucorum*.

Insects are thermotropic organisms, as changes in external environmental temperatures would directly cause changes in their body temperatures and metabolic rates [15]. High temperatures affect the water retention system of insects by making cells lose water more easily, causing changes to the microenvironment of cuticles, and making them more susceptible to insecticide adversity [16, 17]. Also, lower or higher temperatures, damage the nucleic acids, proteins and other biological macromolecules in insects to cause metabolic disorders [18]. Heat shock proteins have been reported to be involved in the protection of insects against stress and are highly expressed when insects are exposed to stress temperature environment [19]. Detoxification enzymes have also been shown to undergo differential expressions, to influence the susceptibility of insects to some insecticides at different temperature. For example, changes in the detoxification enzymes activities in *Bemisia tabaci*, in response to temperature variations, was reported to have influenced its susceptibility to insecticides [20]. Also, the metabolic-related P450 gene in *Drosophila melanogaster*, was reported to have been down-regulated after high-temperature pre-exposure [21]. Previous work in our laboratory showed that the responses in *A*. *lucorum* to a positive TEI (imidacloprid) at 35°C involved more detoxification enzymes and stress response transcripts than at 15 and 25°C, which was the case for a negative TEI (*β*-cypermethrin) at 15°C [22]. UDP-glucuronyltransferase (UGT) and heat shock protein (HSP) transcripts were also heavily involved in the temperature effect of this pests to insecticides [22]. In our research, the sensitivity of *A*. *lucorum* to hexaflumuron (positive TEI) at 15°C and to *β*-cypermethrin (negative TEI) at 35°C also increased with increase in pre-exposure time. However, the physiological and biochemical changes involved, especially the detoxification enzymes and HSPs, need to be further studied.

Results of this study also showed that, compared with the shorter pre-exposure time, the susceptibility of *A*. *lucorum* to hexaflumuron (at 15°C) and *β*-cypermethrin (at 35°C) increased more obviously after 4 h pre-exposure treatment (Tables 1 and 2). For example, the LC_50_ values of hexaflumuron (at 15°C) under 2 h and 4 h pre-exposure treatment were 1459.98 mg/L and 1237.15 mg/L respectively, but were 603.53 mg/L, 294.76 mg/L and 237.40 mg/L under 8 h, 12 h and 24 h pre-exposure treatment respectively. The shorter pre-exposure time to temperatures may have delayed the increasing of the sensitivity of *A*. *lucorum* to the insecticides, thereby exhibiting the hormesis effect [23–26].

In summary, our results showed that the toxicities of positive TEI (hexaflumuron) and negative TEI (*β*-cypermethrin) to *A*. *lucorum* were significantly enhanced at insensitive temperatures respectively. The TCs of hexaflumuron and *β*-cypermethrin decreased, while phoxim (non TEI) did not change significantly, when the pre-exposure time was increased. Based on the results, we conclude that the duration of the acclimation time of insects, before their exposures to insecticides, may reduce the degree of TE which depends on the increased toxicity of TEI at insensitive temperatures, rather than at sensitive temperatures. These studies can also guide the precise application of insecticides in the field, for example, suggesting that the positive TEI hexaflumuron is more effective in the early morning than at dusk at the same lower temperatures, in addition to being used at high temperatures. This is because the TE of hexaflumuron is reduced, after *A*. *lucorum* have been pretreated with low temperatures overnight. While the negative TEI β-cypermethrin is recommended for use at low temperatures, but when at the same higher temperature, the control effect on green bugs is better in the afternoon than in the morning after a high temperature pretreatment at noon. The mechanisms underlying the influence of pre-exposure time of *A*. *lucorum*, to the biological activities of the three insecticides with different TEs, may be related to physiological functions such as that of detoxifying enzymes and hormesis effect in the insect. However, this also requires further studies.
